# Changes in Fruit and Vegetable Consumption and Leisure Time Physical Exercise after a Citizen Science-Based Worksite Health Promotion Program for Blue-Collar Workers

**DOI:** 10.3390/ijerph192013652

**Published:** 2022-10-21

**Authors:** Sophie van der Feltz, Henk F. van der Molen, Lisa Lelie, Carel T. J. Hulshof, Allard J. van der Beek, Karin I. Proper

**Affiliations:** 1 Department of Public and Occupational Health, Amsterdam Public Health Research Institute, Amsterdam UMC, Vrije Universiteit Amsterdam, 1007 MB Amsterdam, The Netherlands; 2 Department of Public and Occupational Health, Amsterdam Public Health Research Institute, Coronel Institute of Occupational Health, Netherlands Center for Occupational Diseases, Amsterdam UMC, University of Amsterdam, 1100 DD Amsterdam, The Netherlands; 3 Centre for Nutrition, Prevention and Health Services, National Institute for Public Health and the Environment, 3721 MA Bilthoven, The Netherlands

**Keywords:** blue-collar workers, construction workers, worksite health promotion program, citizen science, effect evaluation

## Abstract

Blue-collar workers have, on average, poorer health than white-collar workers. Existing worksite health promotion programs (WHPPs) are often not successful among blue-collar workers. This study evaluates the effect of the Citizen Science-based WHPP on the targeted lifestyle behaviors among construction workers. The data of 114 participants were retrieved from questionnaires before (T0) and after (T1) the WHPP. Outcome measures were mean and categorical changes in daily fruit and vegetable intake and weekly leisure time physical exercise. Changes were tested using Wilcoxon signed rank tests and McNemar tests. No statistically significant changes were found between T0 and T1. In total, 73.7% of the participants felt involved in the WHPP. Changes in the outcome measures were not significantly different between subgroups based on age, nor in subgroups based on feelings of involvedness. The low intensity of the developed program could be an explanation for this lack of significant change. Future studies using the Citizen Science approach in an occupational setting should aim at developing a more intensified program and should test its effectiveness by comparing changes in a (randomized) controlled trial.

## 1. Introduction

On average, blue-collar workers have a poorer health than white-collar workers [1,2,3]. Next to their often-poorer physical working conditions [2,4,5], blue-collar workers, who are mostly lower educated, have poorer lifestyle behaviors than white-collar workers with a higher educational level. For example, they have a less healthy diet and less leisure time physical activities, while they smoke more often compared to white-collar workers [6,7,8,9]. As the adverse health effects of unhealthy lifestyle behaviors are evident [10,11,12,13,14], the promotion of healthy lifestyle behaviors among the target group of blue-collar workers is necessary to achieve health gains.

An appropriate setting to promote the health and lifestyle behaviors of workers, including blue-collar workers, is the worksite [15,16,17,18,19]. Worksite Health Promotion Programs (WHPPs) aim to improve the lifestyle choices of workers and thereby their health in the long term. Although recent individual participant data analyses did not reveal differences in the effectiveness of WHPPs across socioeconomic groups of workers [20,21], other studies showed that WHPPs were less successful among blue-collar workers than among white-collar workers [22]. One of the explanations is that WHPPS are often cognition-based, while blue-collar workers are more practical minded [23,24]. As the WHPPs often do not meet the skills, knowledge and needs of blue-collar workers [25,26], these workers tend to comply less to WHPPs and drop out of the program early, which can severely decrease the impact of the WHPP [27,28,29].

An alternative approach to improve the lifestyle behaviors of blue-collar workers is through participatory methods. A review has shown that involving participants in the design and implementation of a WHPP can increase reach and compliance [30]. One participatory method is the Citizen Science approach, which is defined as “the general public engagement in scientific research activities when citizens actively contribute to science either with their intellectual effort or surrounding knowledge or with their tools and resources” [31]. In a Citizen Science approach, researchers work closely together with the target population to create a program using scientific knowledge, as well as knowledge of the target group. Previous Citizen Science programs have shown promising results [32,33,34,35] and by following this method, the WHPP could be tailored more specifically to the target group.

There are two main ways in which the target population can be actively involved in Citizen Science-based research: contributory Citizen Science and co-created Citizen Science. Contributory Citizen Science is performed within a top-down structure, in which the engagement of citizens in the research is limited. Co-created Citizen Science is structured bottom-up, engaging citizens in (almost) all steps of the research process [36,37,38]. Recently, the co-created Citizen Science approach has been used for the development of a WHPP within a work environment with a high proportion of blue-collar workers [39]. In co-creation, the employees, stakeholders such as Human Resource and management and the researchers identified the health issues and lifestyle behaviors to target. To our knowledge, this was the first time that the co-created Citizen Science approach has been applied with the aim to develop and implement a WHPP. However, the effectiveness of such a co-created Citizen Science-based WHPP has yet to be assessed. Therefore, the current study aims to evaluate the changes in targeted lifestyle behaviors after the co-created Citizen Science-based WHPP among workers in a construction company with a high proportion of blue-collar workers.

## 2. Materials and Methods

### 2.1. Study Design and Medical Ethics

Data were retrieved through questionnaires that were conducted before (T0) and after (T1) the implementation of the Citizen Science-based WHPP (i.e., pre-post study design). The Medical Ethics Committee of VU University Medical Center (Amsterdam, The Netherlands) has declared that ethical approval was not necessary for this study. All respondents signed informed consent forms before participation.

### 2.2. Study Population

**Figure 1:**
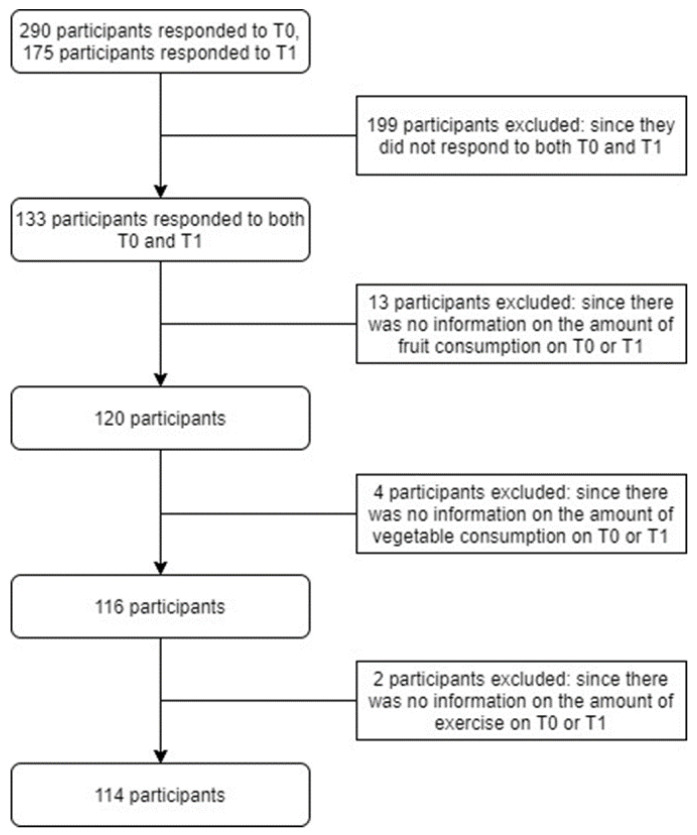
Flowchart of study population.

The study population included workers from a Dutch construction company aged between 18 and 67 years. The company was recruited from the network of the researchers and selected because of its relatively high proportion of blue-collar workers and its willingness to participate [39]. The company delivers services related to construction, restoration, renovation and maintenance of buildings. The company had 309 employees, of which 133 were blue-collar workers and 176 were white-collar workers. Participation to the co-creation of the WHPP was voluntary and no additional motivation strategies were used [39]. When the developed WHPP was implemented, the company made participation obligatory for its employees, but respondence to the questionnaires was voluntary. In total, 133 workers that participated in the Citizen Science-based WHPP responded to the questionnaires on both T0 and T1. Respondents were excluded from this study when data were missing on the outcome measures as defined by vegetable or fruit consumption (n = 17) or physical exercise (n = 2), either on T0 or T1. Finally, 114 participants were included in the analyses of this study (Figure 1).

### 2.3. The Citizen Science-Based WHPP Intervention

The company wide implemented WHPP was developed using the Citizen science approach. Employees, managers, HR advisors and the prevention team of the company were included in five steps in its development [39]. During the first step, semi-structured individual interviews were conducted with employees in order to identify barriers and facilitators for health improvement and feasible elements of Citizen Science. The results from these interviews were discussed in the second step during a focus group with the managers, prevention team and HR advisors of the company. During this focus group, the feasible elements of Citizen Science were further discussed and adapted to the worksite and organization. Thirdly, these results were used by experts (in Citizen Science, from the national occupational health institute of the construction industry, in communication of health information to people with low health literacy and in occupational health) to create materials and workshops for the WHPP adapted to and suitable for the company and its employees. In the fourth step, most employees that were interviewed in step 1 participated in a focus group to improve the WHPP materials created by the group of experts. Finally, the WHPP was tested on feasibility and the extent to which it reflected the needs of all employees. The pilot test was performed amongst the interviewees and two employees that were not involved in the previous process, who all gave oral feedback on the materials afterwards [39].

Finally, the developed WHPP consisted of two “toolbox” sessions covering different health behavioral themes. A toolbox is an existing intervention instrument in the construction sector and consists of interactive and educational meetings about safety at work. Thus, this intervention type was familiar to the current participants. The WHPP toolboxes were organized in an interactive manner with 5–20 participants lasting around 30–45 min per session. The first toolbox discussed physical exercise during leisure time, working postures and rest during work. The second toolbox covered a healthy diet, in particular focusing on fruit and vegetable consumption.

During the toolbox sessions, participants received information via a factsheet and an interactive presentation. Participants were invited to discuss the theme and share their experiences with regards to the theme. Moreover, an idea box was placed on the construction site to anonymously give health promoting suggestions.

### 2.4. Data Measurement

Data were gathered in October–November 2019 (T0) at the start of the first toolbox and in February–March 2021 (T1), some months after the second toolbox took place. At T0, a questionnaire on paper was used to gather the data and, due to the COVID-19 pandemic, an online questionnaire was used at T1. Questions were based on validated questionnaires and previous studies [40,41]. The outcome measures were fruit and vegetable consumption and leisure time physical exercise, as these were the topics addressed in the two executed toolboxes.

#### 2.4.1. Fruit and Vegetable Consumption

First, respondents were asked how many days per week they consumed fruit and vegetables. Then, they were asked how many pieces of fruit and serving spoons of vegetables they consumed on average on the days that they consumed those products. In addition to the daily fruit consumption in pieces and vegetable consumption in serving spoons, respectively, a dichotomous variable was created based on the Dutch guidelines for fruit and vegetable consumption. For this, a cut-off point was used for <2 versus ≥2 pieces of fruit and <5 versus ≥5 serving spoons of vegetables per day [42].

#### 2.4.2. Leisure Time Physical Exercise

Respondents were asked to specify which types of sport they performed and on how many days per week they performed each sport. Moreover, they could indicate the intensity of these sports (light, moderate, heavy). Next, for the number of days per week of exercise of at least moderate intensity, a dichotomous variable was created based on the toolbox goal, i.e., performing at least moderate exercise in leisure time for ≥2 times per week.

#### 2.4.3. Other Variables

Questions about body height (at T0), body weight, age, perceived health, intention to live healthier and influence of work environment on health were asked. Body height and weight were used to calculate body mass index (BMI) on T0 and T1. These variables were used to gain additional insight into the characteristics of the study population.

### 2.5. Statistical Methods

Descriptive statistics were used to gain insight into the characteristics of the study population. Continuous variables were presented by their median and interquartile range (IQR), except for age, which was presented by its mean and standard deviation (SD). Categorical variables were presented by frequencies and relative percentages. Continuous changes were presented by their mean and SD. Furthermore, categorical changes in the daily consumption of fruit and vegetables and number of weekly days of exercise between T0 and T1 were calculated. These changes were categorized into: “decreased”, “unchanged” or “increased” and subsequently presented in graphics to show the proportions of change for each behavioral outcome measure of the whole population. Moreover, these changes were presented for different subgroups by age (younger than average versus average age and older) and the degree of active involvement in the toolbox (not or neutrally involved versus involved).

Since none of the data were normally distributed, the Wilcoxon signed rank test was performed to test the statistical significance of the differences between T0 and T1 of the continuous outcome variables. The McNemar test was used to statistically test the differences between T0 and T1 of the dichotomous outcome variables. Finally, the Chi-squared test was used to test for significance in the differences between proportional changes in the presented subgroups. For all tests, a *p*-value of ≤0.05 was used.

Statistical analyses were executed using SPSS Statistics 26.

## 3. Results

### 3.1. Baseline Characteristics

Table 1 shows the descriptive baseline characteristics of the study population (n = 114). The mean age of the study population was 43.6 (SD 11.5) years. Almost nobody adhered to the Dutch guideline recommendations concerning vegetable intake (96%) and around two thirds (68%) did not meet the Dutch guideline recommendations as to fruit intake. Regarding physical exercise, around two thirds (67%) did meet the toolbox goals of performing exercise in leisure time at least twice per week. Some participants did not report how intensive their sport was (n = 7); these values were completed based on the intensity level of their sport in the other questionnaire or the intensity level that other participants attributed to the same sport. Almost half of the population was either overweight or obese (48.6%). The vast majority perceived their health as (very) good or excellent (>90%); the majority (54%) believed they already lived healthy, while 38.1% had the intention to live healthier.

### 3.2. Changes of Fruit and Vegetable Consumption and Physical Exercise between T0 and T1

The mean change in vegetable consumption was −0.01 spoons per day with a standard deviation (SD) of 1.17. For fruit consumption, the mean change was −0.03 pieces per day (SD 0.60). The mean change in the frequency of physical exercise was 0.26 per week (SD 2.01). The Wilcoxon signed rank did not show any statistical significance in the continuous changes. Additionally, the McNemar test performed on the dichotomous outcomes showed no significant changes between T0 and T1.

**Figure 2:**
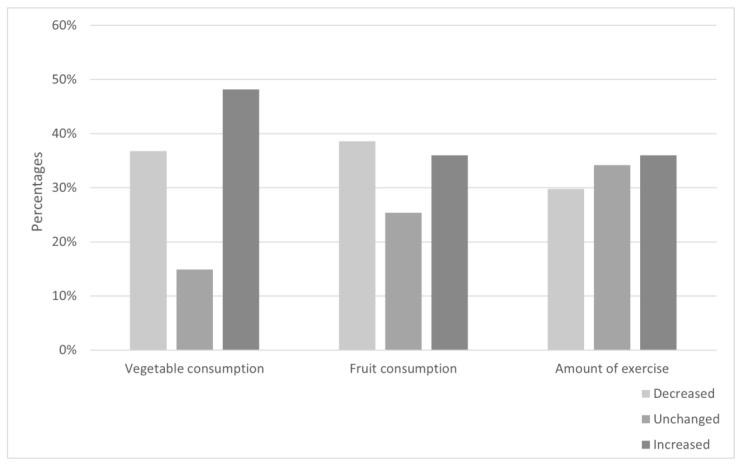
Percentages of categorical change in vegetable and fruit consumption and exercise between T0 and T1.

Figure 2 shows the proportions of changed lifestyle behaviors: decreased, unchanged or increased for amount of fruit or vegetable consumption and frequency of physical exercise. Regarding vegetables, 37% had a decreased consumption, 15% had an unchanged consumption and 48% had an increased consumption. Regarding fruit, 39% had a decreased consumption, 25% had an unchanged consumption and 36% had an increased consumption. Regarding physical exercise, 30% had a decreased frequency of exercise, 34% had an unchanged frequency of exercise and 36% had an increased frequency of exercise.

**Figure 3:**
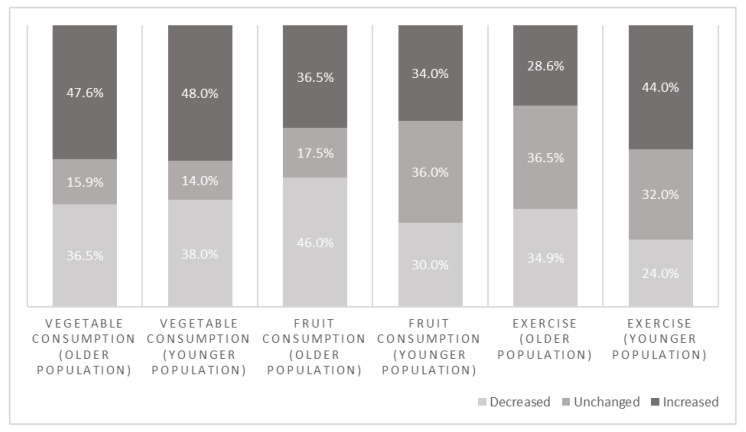
Percentages of categorical change in lifestyle behaviors between T0 and T1 within two age groups.

Figure 3 shows the categorical changes per lifestyle behavior for two subgroups based on age. The older group aged 43.6 years and older contained 63 respondents and the younger group being younger than the average of 43.6 years contained 50 respondents. Some differences in changes between the subgroups are visible, but none of these differences appeared to be significant as tested by the Chi-squared test.

**Figure 4:**
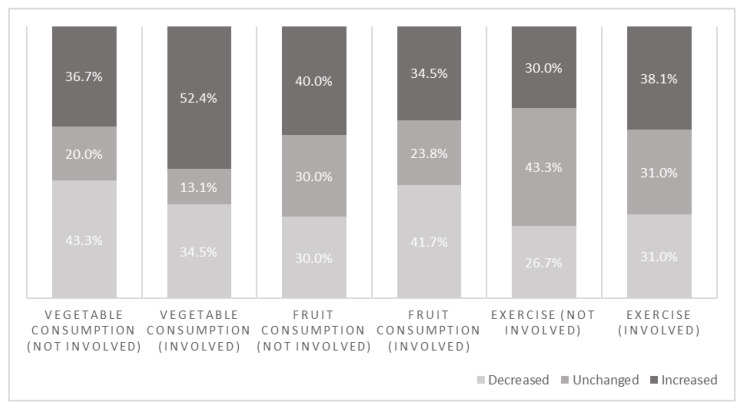
Percentages of categorical change in lifestyle behaviors between T0 and T1 within involvement-based subgroups.

Figure 4 shows the changes per lifestyle behavior for two subgroups based on involvement in the WHPP (i.e., “not involved or neutral” and “involved”). The group that did not feel involved or was neutral consisted of 30 respondents (26.3%), while the group that did feel involved consisted of 84 respondents (73.7%). Again, none of the differences in changed lifestyle behaviors between the subgroups were significant.

## 4. Discussion

This study evaluated changes in lifestyle behaviors before and after a Citizen Science-based WHPP in a population with a high proportion of blue-collar workers. Using a pre-post study design, no significant changes in fruit and vegetable consumption and physical exercise were found. The majority of the participants did show changes regarding the lifestyle factors, but they were both negative (decrease) and positive (increase) and did not point in one clear direction. However, the vast majority of the population did feel (very) involved in the Citizen Science based-WHPP. When performing subgroup analyses by age and involvement in the WHPP, no significant differences within the lifestyle changes were found. This is inconsistent with the literature, which found that WHPPs often show a higher effect in younger populations [22].

Even though previous Citizen Science programs showed promising results [32,33,34,35], the effectiveness of applying this approach to conduct a WHPP is not supported by the findings of the current study. These results are in consistency with a previously conducted meta-analysis, which concluded that in all socioeconomic groups, most WHPPs did not show an effect [20]. Studies that did find WHPPs to be effective reported small effects sizes [22,43]. Moreover, the lack of behavioral changes might be explained by the low intensity of the developed program. The hosting of two toolboxes of approximately 30–45 min each is presumably not enough to change lifestyle behaviors. A systematic review found that the effect of WHPPs is generally low and is partly determined by intensiveness of the program, concluding that more intensive programs with weekly contacts showed more effect [22]. Similar conclusions about importance of intensiveness were drawn by a meta-analysis, which found that WHHPs consisting of more than five sessions and that were organized on an individual level appeared to be more effective [21]. Another fact to consider is that due to a lack of time in the current occupational setting, it is questionable to what extent the development of the WHPP was in accordance with the Citizen Science approach [39]. The employees were only available for a limited amount of time to contribute to the development of the program and researchers had to give more guidance than planned beforehand. Furthermore, the COVID-19 pandemic had an impact on the implementation of the WHPP, as the second toolbox sessions were postponed for several months and the initially planned third toolbox sessions about goal setting were not conducted. The study period was therefore extended in time, but since there was much more time between the toolbox sessions, it is plausible that at least the effect of the first toolbox weakened over time. The COVID-19 pandemic might also have had an influence on the lifestyle behaviors of the current study population. For example, the closing of gyms and cancelling of group sports activities might partly have caused the observed decrease in physical exercise for some workers. Moreover, research has found that the pandemic has led to unhealthy dietary habits, such as eating and snacking more [44,45,46].

A weakness of this study includes that there was no control group, which made it impossible to compare the results with those in an equivalent group that did not participate in the WHPP. Another weakness is the self-report measurements of the outcome variables. Next to the use of questionnaire, which are known to be less valid than objective instruments, vegetable consumption was measured by the amount of serving spoons. Not only do serving spoons come in different sizes, but it also depends on the kind of vegetable how much one serving spoon contains. For example, one serving spoon of lettuce weighs and contains a lot less than a serving spoon of cooked spinach. Therefore, more objective measurements of vegetable consumption would have been desirable and is recommended for future studies. Furthermore, it is noteworthy that the toolboxes were obligatory and almost all workers in the company participated, but less than half of the participants filled in both questionnaires. In addition to a lower response than hoped for, it could have led to selection bias and an overestimation of the results if mostly the more motivated participants responded. Moreover, no data were available on the type of work conducted by the participants. Therefore, we do not know what the exact proportion of blue-collar workers was in the current study population.

Future Citizen Science-based WHPPs should try to develop a more intensified program in order to see whether this improves the effectiveness. Moreover, a randomized controlled study design or a design with at least an equivalent control group is recommended to evaluate the effect of a WHPP that results from a Citizen Science approach and to analyze whether the Citizen Science approach can lead to more effective WHPPs. Finally, the effect of the program on different subpopulations should also be studied further, since our findings are inconsistent with the literature. Tailoring the program to different subgroups might improve the effectiveness as well.

## 5. Conclusions

Even though no significant changes in the targeted lifestyle behaviors were found for the Citizen Science-based WHPP in construction workers, there was a high proportion of participants that felt (very) involved in the WHPP. This encourages more elaborate research into Citizen Science-based WHPPs. Intensifying the final program developed by the Citizen Science approach might improve its effectiveness.

## Figures and Tables

**Table 1:** Characteristics of the study population at baseline ^1^.

Variables	Category	N (%)
Age ^2^	Mean ± SD	43.6 ± 11.5
Body Mass Index (BMI) ^3^	Underweight	2 (1.9)
	Healthy weight	52 (49.5)
	Overweight	42 (40.0)
	Obesity	9 (8.6)
	Mean ± SD	24.93 ± 3.19
Vegetable Consumption	≥5 serving spoons per day	5 (4.4) ^4^
	<5 serving spoons per day	109 (95.6)
	Median ± IQR serving spoons per day	2.14 ± 1.57
Fruit Consumption	≥2 pieces per day	36 (31.6)
	<2 pieces per day	78 (68.4)
	Median ± IQR pieces per day	1.43 ± 1.29
Physical exercise	≥2 times per week (moderate) intensive exercise	78 (68.4)
	<2 times per week (moderate) intensive exercise	36 (31.6)
	Median ± IQR exercise per week	2.00 ± 2.13
Perceived health ^5^	Excellent	8 (7.3)
	Very good	26 (23.9)
	Good	67 (61.5)
	Poor	8 (7.3)
	Very poor	0 (0.0)
Intention to live healthier ^6^	Never thought about it	4 (3.5)
	Thought about it, but does not know yet	5 (4.4)
	Intention not to (start to) live healthy	0 (0.0)
	Intention to live (more) healthy	43 (38.1)
	Believes they already live healthy	61 (54.0)
Influence work environment on health ^7^	Very good influence	0 (0.0)
	Good influence	23 (20.5)
	No influence	48 (42.9)
	Bad influence	40 (36.7)
	Very bad influence	1 (0.9)

^1^ Total study population: n = 114. ^2^ Number of missing in age: n = 1 ^3^ Number of missing in BMI: n = 9 in T0 and n = 22 in T1. ^4^ Numbers are presented as frequencies with relative percentages between brackets for the categorical outcome variables and they are presented as medians ± IQR for the continuous outcome variables. ^5^ Number of missing in opinion own health: n = 5 in T0. ^6^ Number of missing in intention to live healthier: n = 1 in T0. ^7^ Number of missing in influence work environment on health: n = 2 in T0.

## Data Availability

All data generated or analyzed during this study are included in this published article.
